# New data on rotifers (Rotifera) from European waterbodies

**DOI:** 10.3897/BDJ.14.e177185

**Published:** 2026-03-03

**Authors:** Nataliia S Iakovenko, Dzmitry Lukashanets, Jolanta Ejsmont-Karabin, Irena Bielańska-Grajner, Eleonora Ovander, Diego Fontaneto, Veselka Tsavkova, Luchezar Zlatev Pehlivanov, Karel Janko

**Affiliations:** 1 University of Ostrava, Ostrava, Czech Republic University of Ostrava Ostrava Czech Republic; 2 Institute of Animal Physiology and Genetics, Libechov, Czech Republic Institute of Animal Physiology and Genetics Libechov Czech Republic; 3 Schmalhausen Institute of Zoology, Kyiv, Ukraine Schmalhausen Institute of Zoology Kyiv Ukraine; 4 Marine Research Institute, Klaipeda University, Klaipeda, Lithuania Marine Research Institute, Klaipeda University Klaipeda Lithuania; 5 Nencki Institute of Experimental Biology, Polish Academy of Sciences, Warsaw, Poland Nencki Institute of Experimental Biology, Polish Academy of Sciences Warsaw Poland; 6 University of Silesia, Katowice, Poland University of Silesia Katowice Poland; 7 National Research Council of Italy, Verbania Pallanza, Italy National Research Council of Italy Verbania Pallanza Italy; 8 Institute of Biodiversity and Ecosystem Research Bulgarian Academy of Sciences, Sofia, Bulgaria Institute of Biodiversity and Ecosystem Research Bulgarian Academy of Sciences Sofia Bulgaria; 9 Institute of Animal Physiology and Genetics AS ČR, v.v.i., Liběchov, Czech Republic Institute of Animal Physiology and Genetics AS ČR, v.v.i. Liběchov Czech Republic

**Keywords:** Pararotatoria, Ploima, Collothecaceae, Flosculariaceae, Bdelloidea, aquatic habitats, biogeography

## Abstract

**Background:**

Rotifers (Rotifera) are microscopic pseudocoelomate animals that represent one of the key components of plankton, periphyton and benthos communities in aquatic ecosystems. As members of the zooplankton, they play an important role as primary consumers of phyto- and bacterioplankton, serve as a food source for fish larvae and act as indicators of water quality. Although rotifers have been studied in Europe for over three centuries, they remain an important subject of zoological research, as their ecological characteristics and biodiversity in several European countries are still insufficiently understood. This is particularly true for bdelloid rotifers, whose study is complicated by the impossibility of reliable identification in fixed material. The availability of georeferenced data on rotifer occurrences, coupled with environmental parameters, is of great importance for such studies, yet such data on the GBIF platform are not complete for many aquatic rotifer species.

**New information:**

We present new georeferenced data on rotifers in all major types of European freshwater and brackish inland waterbodies: lotic (rivers, streams, brooks, waterfalls, canals), lentic (lakes, ponds, swamps, bogs, marshes, puddles, cryoconite holes and other temporary waterbodies) and transitional (estuaries). The littoral zone of the Black Sea and the southern coast of the English Channel (La Manche) are represented as marine habitats in this dataset. Twenty-five protected areas were surveyed during this study.

The survey was conducted from 1976 to 2021. In total, we added to GBIF 4,862 new occurrences of Phylum Rotifera, including the classes Eurotatoria (subclasses Monogononta and Bdelloidea) and Pararotatoria. The dataset substantially expands the information on the distribution of still inadequately studied bdelloid rotifers (Bdelloidea) available through GBIF (1,514 new occurrences are added). We focused our study on aquatic communities that have received less attention than planktonic ones, such as periphytic, benthic and psammic rotifers and commensals on aquatic invertebrates. We also added new data on rotifers in waterfalls (6 species), temporary waterbodies (153 taxa of species rank) and tree hollows (5 species). Three species amongst those recorded are new to the fauna of Ukraine: *Lecane elsa* Hauer, 1931, *Lindia pallida* Harring & Myers, 1922, *Paradicranophorus aculeatus* (Neizvestnova-Zhadina, 1935).

## Introduction

The study of rotifers in European waters began as early as the first microscopic research (1703–1714), following the invention of a new type of microscope by Antonie van Leeuwenhoek (Koste and Hollowday 1993). Nethertheless, even after more than three centuries, the diversity and ecology of rotifers from many European regions and ecosystems remain insufficiently studied, as do the distribution and taxonomy of some groups, such as Gnesiotrocha and Bdelloidea. Despite the vast body of literature on rotifer fauna, ecology, taxonomy and systematics published since the early monographs by Ehrenberg (1838) and Hudson and Gosse (1886), Hudson and Gosse (1889), summarised in identification keys and surveys of 20^th^ century (Voigt 1957, Ruttner-Kolisko 1972, Koste 1978, Dumont 1983, Nogrady et al. 1995, De Smet 1996, De Smet and Pourriot 1997) and the beginning of 21^st^ century (De Smet 2002, Nogrady and Segers 2002, Segers 2002, Wallace et al. 2006, Segers 2007, Fontaneto et al. 2008, Jersabek et al. 2012, De Smet 2015, Wilke et al. 2019, Ferrari et al. 2023), significant gaps in our knowledge of European rotifers persist.

Most records of aquatic rotifer occurrences published before the 2000s were not georeferenced, necessitating additional work to make these data suitable for modern biogeographic and ecological analyses. Researchers' attention was largely focused on planktonic rotifers, given their importance as a food source for fish and as indicators of water quality (Lubzens et al. 1989, Onandia et al. 2021, Karpowicz et al. 2025). In contrast, taxa inhabiting periphyton, benthos and psammon received comparatively less attention (Bielańska-Grajner 2001, Duggan 2001, Wallace et al. 2021, Denys and De Smet 2023). The development of these studies has been hampered by the lack of standardised quantitative methods for collecting and preserving specimens, particularly those with soft, easily deformable bodies. Research on rotifers in activated sludge from wastewater treatment plants has also remained limited (Kutikova 1984, Seaman et al. 1986, Kocerba-Soroka et al. 2013). Similarly, investigations of rotifers in temporary and small-volume waterbodies, such as tree holes (Hauer 1924) and cryoconite holes on Svalbard (De Smet 1993), are far from complete.

Bdelloid rotifers (Rotifera, Eurotatoria, Bdelloidea) remain understudied both globally and within Europe. Seminal works by Voigt (1957), Bartoš (1959), Rudescu (1960) and Donner (1965) left many gaps concerning the distribution and ecology of aquatic bdelloids on the continent. The research group led by Profs. C. Ricci (now deceased) and G. Melone made substantial contributions to the taxonomy and ecology of this obligately parthenogenetic clade (Ricci 1987, Melone and Ricci 1995, Ricci and Melone 2000, Ricci and Balsamo 2001, Ricci 2016). Their work opened new avenues for studying aquatic bdelloids through modern approaches, such as laser confocal and scanning electron microscopy, molecular ecology and phylogenetic analysis (Melone and Fontaneto 2005, Fontaneto et al. 2007a, Fontaneto et al. 2007b, Leasi et al. 2010). Contemporary studies of bdelloid diversity now require these methods, for example, for species delimitation.

Rotifer research in Eastern Europe began in the mid-19^th^ - the beginning of 20^th^ century (Stepanov 1886, Skorikov 1896, Voronkov 1907). The two most comprehensive monographs on Rotifera from the region were published by Kutikova (Kutikova 1970, Kutikova 2005), who noted that the fauna of certain areas, such as the European North and East, remained insufficiently investigated. Faunistic surveys of the last 20 years have covered many areas of Ukraine and Belarus, often re-analysing historical data in light of modern taxonomy (Galkovskaya and Molotkov 2001, Galkovskaya et al. 2001, Iakovenko and Ovander 2011, Iakovenko et al. 2011, Ovander et al. 2011a, Ovander et al. 2011b, Gromova et al. 2019, Lukashanets 2018). Nevertheless, this work remains incomplete and requires further studies employing morphological, molecular and SEM-based approaches.

The early studies of Rotifera in Poland were initiated by Wierzejski (1891), Bloedorn (1912), Pawłowski (1958), Wiszniewski (1933), Wiszniewski (1934). The most recent monographs on Polish rotifers (Bielańska-Grajner et al. 2004, Bielańska-Grajner et al. 2013) summarise current knowledge of the taxon in the region, but still leave many gaps in our understanding of rotifer diversity and distribution in Poland.

The objective of this study is to further elucidate our knowledge of Rotifera in European waters, with particular emphasis on diversity and distribution of rotifer species in Eastern, Northern and Southern Europe. We also aimed to address existing gaps in the documentation of rotifer fauna within protected European areas. We achieved it by compiling and publishing new georeferenced records of Rotifera from European waterbodies, with particular emphasis on less studied habitats, such as swamps, temporary waterbodies, cryoconite holes and tree hollows. The data presented in this paper should provide a valuable foundation for future ecological and biodiversity research on aquatic organisms inhabiting both freshwater and marine environments across Europe.

## Project description

### Title

ROTISFERA 'Global patterns of microinvertebrate distribution: does diversity decrease poleward in rotifers (Rotifera, Bdelloidea)?’ NDICI-GEO-NEAR/2022/434-092-0068

### Personnel

Dzmitry Lukashanets, Nataliia Iakovenko

### Study area description

The project encompasses all major regions of the Earth.

### Design description

To address the project's aims, we analysed more than 961 samples collected from 1976 until 2021 in all major types of European waterbodies in 16 countries. The data cover altitudes from 0 to 1398 m of altitude. Samples were analysed using methods of classical taxonomy (analysis of rotifer morphology using light microscopy) and, in some cases, confirmed by molecular and SEM methods. The analysis resulted in a dataset with 4,862 rotifer species occurrences, 68.8% of which belong to Monogononta (Ploima, Collothecaceae and Flosculariaceae) and 31.1% to Bdelloidea.

### Funding

European Union.

## Sampling methods

### Study extent

**Figure 1.**
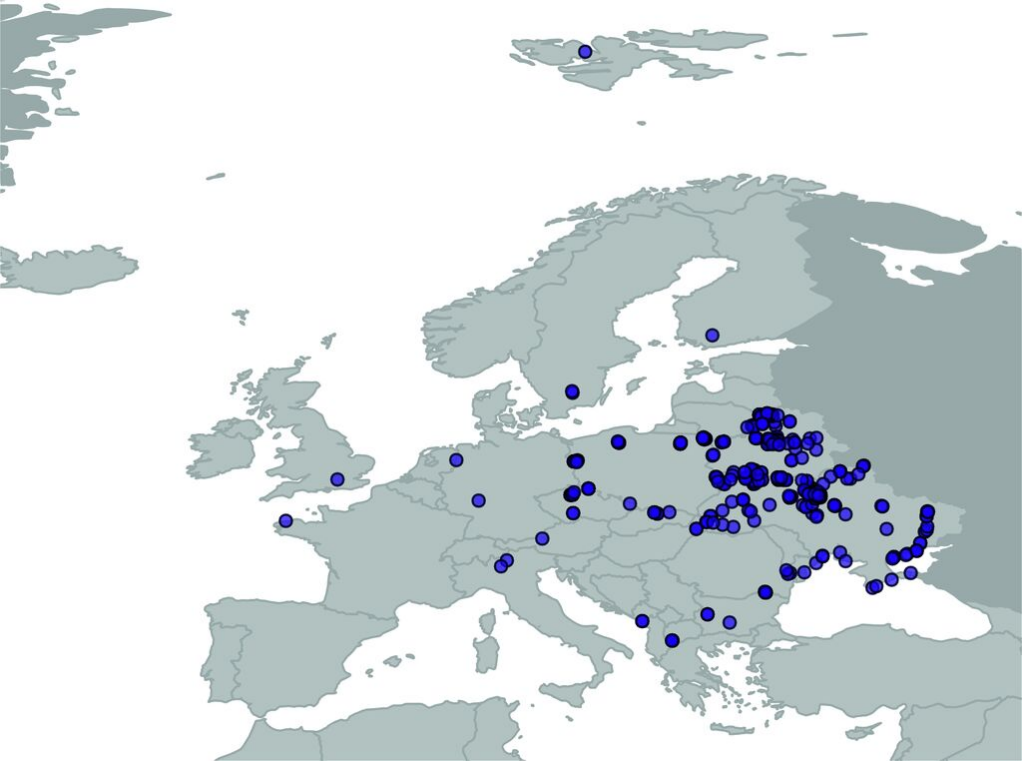
All sampling sites included in the dataset.

**Figure 2.**
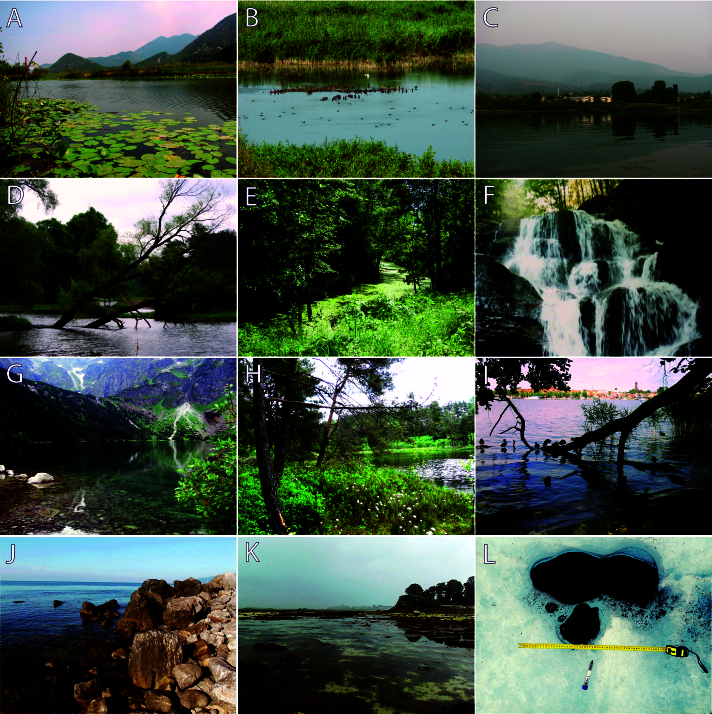
Examples of waterbodies studied in Europe: A – Lake Skadar (Shkodra), Montenegro; B – Lake Srebarna in the Srebarna Nature Reserve, Bulgaria; C – Lake Prespa, North Macedonia; D – the Irpin' River, Kyiv region, Ukraine; E – the Teteriv River tributary, Kyiv region, Ukraine; F – the Shypit waterfall, Zakarpattia region, Ukraine; G – Lake Morskie Oko, Tatra National Natural Park (NNP), Poland; H – Lake Ślepe, NNP Wigry, Poland; I – Lake Mikołajskie, Warmian-Masurian Voivodeship, Poland; J – the Black Sea coast at Cape Martian Natural Reserve, Ukraine; K – the sea littoral of La Manche (the English Channel) near Roscoff, France; L – a cryoconite, Svalbard, Norway. Photos: A-C, I-K – N. S. Iakovenko; D, E – V. A. Korneyev; F – M. Ivanov and V. Gnyp; G, H – J. Ejsmont-Karabin; L – K. Janko.

All samples used in the dataset were collected within the framework of several research projects focused on the biodiversity and ecology of aquatic microfauna in Europe Fig. 1. Examples of the waterbodies where rotifers were collected are shown in Fig. 2. The collections were either processed on site during the expeditions and field excursions (basisOfRecord: LivingSpecimen) or preserved for later study in 37% water solution of formaldehyde (basisOfRecord: PreservedSpecimen).

### Sampling description

Sampling was performed using standard hydrobiological methods (Wallace et al. 2006). Apstein nets (20-35 µm) were used for collecting plankton, plastic or glass samplers for collecting benthos, periphyton and psammon. Living samples were transported to the laboratory immediately after the collection in the dark and in thermally isolated vials or identified on site using a mobile laboratory with the 400x total magnification field microscope. Fixation with 37% formaldehyde was used for the preservation of the samples on site. The volume of the sample varied from 1-50 litres concentrated to 250 ml (planktonic samples) to 50-250 ml without concentration (periphyton, benthos and psammon); rotifer densities were recalculated to l^-1^ afterwards. All sampling sites were georeferenced. The environmental parameters (water temperature, pH, oxygen concentration) were measured on site using Hanna Instruments devices (HI8424 portable pH/mV/temperature meter and/or portable oxygen meter), at the time of collection (plankton) or immediately after in the collection vial (periphyton, benthos). Salinity was measured in the laboratory using a benchtop conductivity meter.

### Quality control

All people who identified rotifers are recognised specialists in rotifer diversity and taxonomy. Primarily, first descriptions together with the existing identification keys and reviews on the taxonomy of rotifers were used (Bartoš 1959, Donner 1965, Kutikova 1970, Koste 1978), including the keys of the series "Guides to the Identification of the Microinvertebrates of the Continental Waters of the World, Rotifera" (Nogrady et al. 1995, Nogrady and Segers 2002, De Smet 1996, De Smet and Pourriot 1997). Georeferenced data were checked by placing latitudes and longitudes on a map.

### Step description

1. Microscopic animals were isolated from fresh or 37% formaldehyde-preserved samples using fine glass micro-pipettes in a Petri dish or plankton counting chamber. When necessary, primary samples were concentrated by filtration through a 50 µm net mesh to a final volume up to 50 ml before processing.
2. Rotifers were counted and sorted under a binocular microscope (Olympus SZ61, Olympus SZX10, NR.3 Nikon SMZ1000).
3. Isolated individuals were identified and photographed under a microscope at a total magnification of 400x (Nikon Eclipse Ts2R, NIB-100F inverted microscope, Olympus CX43). To extract the trophi (sclerotised part of the masticatory apparatus), the soft part of the rotifers were dissolved in approximately a 5:1 mixture of water and commercial bleach solution. The isolated trophi were then observed under oil immersion at a total magnification 1,000x. A set of custom needles and whiskers was used to rotate the rotifers to observe the important diagnostic traits (Taylor 2005).
4. Rotifer densities were standardised to 1,000 cm^3^ (l^-1^).

## Geographic coverage

### Description

The dataset includes records collected in 16 European countries (Fig. 1), spanning latitudes from N 79.1 to N 40.9 and altitudes from 0 to 1398 m above sea level:

(i) Austria – Tyrol;

(ii) Belarus – Minsk, Brest, Vitebsk, Grodno, Gomel and Mogiliov region;

(iii) Bulgaria – Silistra, Sofia and Plovdiv oblast, Srebarna Nature Reserve;

(iv) Finland – Kanta-Häme region, Torronsuo National Park;

(v) France – Brittany, coast near Roscoff;

(vi) Germany – Rhineland-Palatinate and Saxony States;

(vii) Italy – Friuli-Venezia Giulia, Lombardy, Piedmont, Tuscany, Umbria regions;

(viii) Montenegro – Lake Skadar;

(ix) Netherlands - Westerwolde Municipality;

(x) North Macedonia – Lake Prespa;

(xi) Norway – Svalbard Archipelago;

(xii) Poland – Lesser Poland, Lower Silesian, Lubusz, Podlaske, Pomeranian, Warmian-Masurian voivodeships;

(xiii) Sweden – Kronoberg region;

(xiv) The Czech Republic – Central Bohemian, South Bohemian, Moravian-Silesian regions;

(xv) The United Kingdom – South East England;

(xvi) Ukraine – Cherkassy, Chernihiv, Dnipro, Donets’k, Ivano-Frankivs’k, Kharkiv, Khmelnyts’k, Kyiv, Lviv, Mykolaiv, Odessa, Poltava, Rivne, Sumy, Ternopil, Volyn, Zhytomyr, Zakarpattia oblast and AR Crimea.

### Coordinates

40.9 and 79.0 Latitude; -3.98 and 37.6 Longitude.

## Taxonomic coverage

### Description

All records in the dataset belong to the Phylum Rotifera, Classes Eurotatoria (Subclasses Monogononta and Bdelloidea) and Pararotatoria. The only recorded species of Pararotatoria is *Seison nebaliae* Grube, 1859, found on the crustacean *Nebalia* sp. from the southern coast of La Manche. Monogonont rotifers (orders Ploima, Collothecaceae and Flosculariaceae) account for the majority of records (68.8%), while bdelloids represent 31.1% of the total records. Species with uncertain affiliation or potentially new for science were indicated as Genus sp., while those differing from the nominal description (mostly potentially new) were designated as cf.

## Temporal coverage

**Formation period:** 1976-2021.

## Usage licence

### Usage licence

Other

### IP rights notes

CC BY 4.0

## Data resources

### Data package title

New data on rotifers (Rotifera) from European waterbodies

### Resource link

Iakovenko N, Lukashanets D, Ejsmont-Karabin J, Bielańska-Grajner I, Ovander E, Tsavkova V, Fontaneto D (2026). New data on rotifers (Rotifera) from European waterbodies. Version 1.10. Marine Research Institute, Klaipeda University. Occurrence dataset https://doi.org/10.15468/p2k9ng accessed via GBIF.org on 2026-01-18.

### Alternative identifiers

https://cloud.gbif.org/eca/resource?r=aquatic_rotifers

### Number of data sets

1

### Data set 1.

#### Data set name

New data on rotifers (Rotifera) from European waterbodies

#### Data format

*.csv

#### Description

This survey is the result of a long-term study of rotifers in European waters conducted by the authors of the dataset. Latitudes covered by the study ranged from N 79.1 to N 40.9 and altitudes from 0 to 1398 m above sea level. All major types of European freshwater and brackish inland waterbodies were studied: lotic (rivers, streams, brooks, waterfalls, canals), lentic (lakes, ponds, swamps, bogs, marshes, puddles, cryoconite holes and other temporary waterbodies) and transitional (estuaries). The littoral zone of the Black Sea and the south coast of La Manche represent marine biotopes in this dataset.

The studies were conducted from 1976 to 2021, during which period more than 961 samples were collected and processed. Sampling covered 16 countries, spanning territories from the Arctic to the Mediterranean and including 25 protected areas (with the necessary permissions). Part of the material from northern Ukraine was collected by E. Ovander in the Chernobyl exclusion zone and nearby regions before the Chernobyl catastrophe (1977-1981). The total number of rotifer records in the dataset is 4,862, including 472 taxa of the species rank (species and subspecies), belonging to 83 genera (67 of Monogononta, 15 of Bdelloidea and 1 of Seisonida) (Iakovenko et al. 2025).

For some samples, the dataset also includes measurements of the environmental parameters (water temperature, pH, oxygen concentration, salinity) along with rotifer densities per litre of volume.

**Data set 1.** 

Column label	Column description
occurrenceID	An unique identifier for the record including the code of the country, year of sampling, ID of the sample and species name.
materialSampleID	An identifier of the sample, from which rotifers were isolated.
eventDate	Date of sampling.
samplingProtocol	Methods of sampling.
geodeticDatum	The geodetic datum, upon which the geographic coordinates given in decimalLatitude and decimalLongitude are based.
decimalLatitude	Latitude in decimal degrees.
decimalLongitude	Longitude in decimal degrees.
verbatimElevation	Elevation above sea level in metres.
continent	The name of the continent where sampling occurred.
country	The name of the country where sampling occurred.
countryCode	ISO 3166-1 alpha-2 country code.
waterBody	The name of the waterbody (lake, river etc.).
locationRemarks	The type of waterbody where sampling occurred.
habitat	A description of the habitat (type of the sample, from which rotifers were isolated).
basisOfRecord	The specific nature of the data record.
scientificName	The full scientific name, with authorship and date information.
individualCount	The number of individuals present at the time of the Occurrence.
identifiedBy	Surname and name of the person who identified the species.
sampleSizeValue	A numeric value for a measurement of the size of a sample.
sampleSizeUnit	The unit of measurement of the size of a sample.
kingdom	The full scientific name of the kingdom in which the species is classified.
phylum	The full scientific name of the phylum in which the species is classified.
class	The full scientific name of the class in which the species is classified.
order	The full scientific name of the order in which the species is classified.
family	The full scientific name of the family in which the species is classified.
genus	The full scientific name of the genus in which the species is classified.
specificEpithet	The name of the species epithet of the scientificName.
infraspecificEpithet	The name of the infraspecies epithet of the scientificName.
taxon rank	The taxonomic rank of the record.
identificationQualifier	A controlled value to express the identifier's doubts about identification.
scientificNameAuthorship	The authorship information for the scientificName
recordedBy	Surname and name of the person who collected the sample, from which rotifers were isolated.

## Additional information

### Overview of taxonomy

The dataset comprises a total of 419 rotifer species (or 472 taxa of the species rank) belonging to 83 genera and 31 families. The order Ploima (subclass Monogononta) accounts for the highest number of occurrences (3,061), representing 65% of all species in the dataset. Bdelloidea contribute 1,514 occurrences (24% of species), followed by the Flosculariacea with 254 occurrences (8%) and the Collothecacea with 32 occurrences (2% of species in the dataset, respectively). The Pararotatoria are represented by a single species, *Seison nebaliae* Grube, 1859, recorded in one occurrence.

### Types of studied waterbodies

The data on rotifer occurrences and taxa of the species rank found in the studied habitats are presented in Table 1.

### Rotifers from conservation-designated habitats

The data on rotifer diversity in the waterbodies of protected regions are presented in Table 2.

### Information on new species for local faunas, rare or less studied species

**Figure 3.**
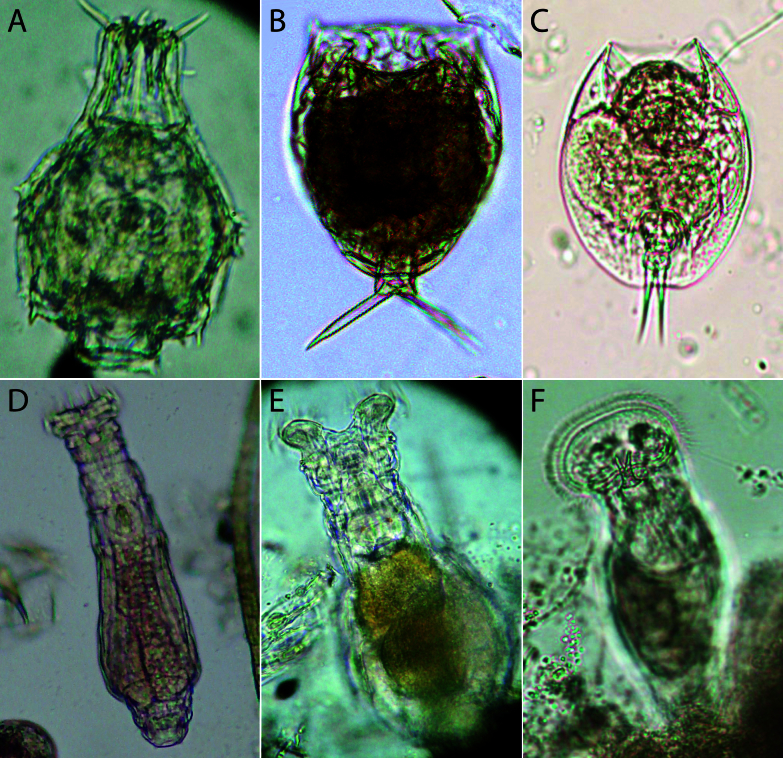
Rare or less studied species of rotifers in Central Europe: **A** – *Dissotrocha spinosa* (Bryce, 1892) from freshwater littoral benthos, Lake Wielkie Gacno, Bory Tucholskie National Nature Park (NNP), Poland; **B** – *Lecane sola* Hauer, 1936 from sphagnum in a dystrophic lake, Lake Suchar III, Wigry NNP, Poland; **С** – *Lepadella rottenburgi* (Lucks, 1912) from sphagnum in a dystrophic lake, Lake Suchar Zachodni, Wigry NNP, Poland; **D** – *Otostephanos monteti* Milne, 1916 from freshwater periphyton (*Chara* sp.), Ostrowite lake, NNP Bory Tucholskie, Poland; **E** – *Philodina rugosa rugosa* Bryce, 1903 from freshwater periphyton, the Wrzosowka River, Karkonosze NNP, Poland; **F** – *Ptygura furcillata* (Kellicott, 1889) from freshwater periphyton, Lake Gilarka, Uscie Warty NNP, Poland. Photos: A, D, E – N. S. Iakovenko; B, C, F – J. Ejsmont-Karabin.

New records in this dataset include three species that are new to the fauna of Ukraine (Table 3). Examples of rare or less-studied rotifer species, along with information on the localities and habitats where they were found, are presented in Fig. 3.

## Figures and Tables

**Table 1.** Number of occurrences and taxa of the species rank found in the studied habitats

Waterbodies	Occurrences	Species and subspecies
Brooks, streams, springs, creeks	205	78
Carst craters	6	5
Channels	93	60
Cryoconite holes	1	1
Estuaries	45	19
Lakes:		
- brackish	26	22
- freshwater	1579	227
- peat (dystrophic)	804	172
Marine habitats	12	8
Ponds	678	139
Reservoirs	42	29
Rivers	358	92
Sewage disposal ponds	25	22
Temporary waterbodies (puddles, ditches etc.):		
- freshwater	492	152
- brackish	3	1
Tree hollows	29	5
Waterfalls	6	6
Wetland (marches, swamps, peat bogs, water meadows)	436	143
Unknown (epibionts)	22	6

**Table 2.** Number of aquatic rotifer occurrences and taxa of species rank, recorded in protected areas (nature reserves and national parks).

Country	Protected region	Number of georeferenced occurrences	Number of species and subspecies
Bulgaria	Srebarna Nature Reserve	600	67
Finland	Torronsuo National Nature Park	5	5
Montenegro	Skadar Lake National Nature Park	3	3
North Macedonia	Ohrid - Prespa UNESCO Transboundary Biosphere Reserve	18	15
Poland	Gorbacz Lake Reserve	17	9
Poland	Karkonosze National Nature Park	33	23
Poland	Tatra National Nature Park	25	20
Poland	Tuchola Forest National Nature Park	124	69
Poland	Warta Mouth National Nature Park	156	88
Poland	Wigry National Nature Park	765	165
Ukraine	Cape Martian Nature Reserve	2	2
Ukraine	Desna–Stara Huta National Nature Park	32	17
Ukraine	Gorgany Nature Reserve	11	11
Ukraine	Holosiiv National Nature Park	11	6
Ukraine	Kaniv Nature Reserve	147	38
Ukraine	Karadag Nature Reserve	1	1
Ukraine	Kam’yani Mohyly (Stone Tombs, part of Ukrainian Steppe Nature Reserve)	22	12
Ukraine	Kremenets Mountains National Nature Park	25	16
Ukraine	Medobory Nature Reserve	6	6
Ukraine	Podil's'ki Tovtry National Nature Park	5	4
Ukraine	Polissia Nature Reserve	21	19
Ukraine	Rivne Nature Reserve	22	17
Ukraine	Shatsk National Nature Park	59	36
Ukraine	Skole Beskids National Nature Park	9	9
Ukraine	Synevyr National Nature Park	14	10

**Table 3.** New for the fauna of Ukraine rotifer species, habitats and localities where they were found

Species	Locality	Habitat type
*Lecane elsa* Hauer, 1931	Volyn' Region, River Nevir Basin, swamp; Zhytomyr region, swamp	Phytophilic plankton (living in the water around aquatic vegetation)
*Lindia pallida* Harring & Myers, 1922	Rivne Region, Lake Bile	littoral lake plankton
*Paradicranophorus aculeatus* (Neizvestnova-Zhadina, 1935)	Kyiv Region, River Teteriv	littoral river plankton
